# Nephrectomy improves the survival of metastatic renal cell cancer patients with moderate to good performance status—results from a Finnish nation-wide population-based study from 2005 to 2010

**DOI:** 10.1186/s12957-021-02308-0

**Published:** 2021-06-28

**Authors:** Lauri Laru, Hanna Ronkainen, Pasi Ohtonen, Markku H. Vaarala

**Affiliations:** 1 Department of Surgery, Medical Research Center Oulu, Oulu University Hospital and University of Oulu, Oulu, Finland; 2 Department of Urology, Oulu University Hospital, PO Box 21, FI-90029 OYS Oulu, Finland; 3 Division of Operative Care, Oulu University Hospital and Medical Research Center Oulu, University of Oulu, Oulu, Finland

**Keywords:** Metastatic renal cell carcinoma, Renal tumor, Population-based, Overall survival, Cytoreductive nephrectomy, Metastasectomy

## Abstract

**Background:**

The purpose of this study was to evaluate the effects of cytoreductive nephrectomy (CN) and metastasectomies on the survival of patients with synchronous metastatic renal cell cancer (mRCC) using real-life, population-based national dataset.

**Methods:**

Nationwide data, including all cases of synchronous mRCC in Finland diagnosed on a 6-year timeframe, based on the Finnish Cancer Registry and complemented with patient records from the treating hospitals, were analyzed. Patients with Eastern Cooperative Oncology Group (ECOG) performance status 3–4 were excluded. Univariate and adjusted multivariable survival analysis were performed, including subgroup analysis for patients with different medical therapies. Nephrectomy complications were also analyzed.

**Results:**

A total of 732 patients were included in the analysis. CN was performed for 389 (53.1%) patients, whereas 68 (9.3%) patients underwent nephrectomy and metastasectomies of all lesions (surgery with curative intent). Median overall survival (OS) for patients who did not undergo nephrectomy was 5.9 (95% confidence interval [CI] = 4.6–7.2) months. Patients who had a CN had a median OS of 16.6 (95% CI = 14.2–19.1, p < 0.001) months, whereas patients who had surgery with curative intent had a median OS of 51.3 (95% CI = 36.0–66.6, p < 0.001) months. The survival benefit of CN and metastasectomies remained significant in all medical therapy subgroups and in both of the applied multivariable statistical models.

**Conclusions:**

Surgical treatment of metastatic renal cell cancer is associated with a significant survival benefit in patients with good and moderate performance status, regardless of the chosen medical therapy.

**Supplementary Information:**

The online version contains supplementary material available at 10.1186/s12957-021-02308-0.

## Introduction

The survival benefit of CN in mRCC is well established in the era of cytokine-based medical therapy [1, 2]. Subsequently, contemporary targeted therapy (TT) with tyrosine kinase inhibitor (TKI) and mammalian target of rapamycin (mTOR) inhibitor medications have shown superior results compared to interferon therapy [3–5]. In 2018, Méjean et al. published their landmark prospective clinical trial on the effect of CN, showing non-inferiority of sunitinib alone, compared to nephrectomy followed by sunitinib in patients with metastatic renal cell carcinoma who were in the Memorial Sloan Kettering Cancer Centre (MSKCC) intermediate- or poor-risk groups [6]. However, multiple retrospective studies continue to support CN as a part of the multimodality treatment regimen in metastatic renal cell cancer in the TT era [7–9].

The biological effect of CN remains somewhat unclear. Several studies support the essential role of immunological mechanisms, such as the primary tumor’s ability to induce apoptosis and impairment of crucial signaling pathways of T lymphocytes [10, 11] and production of pro-inflammatory and T cell inhibitory cytokines and growth factors, promoting metastasis growth [12, 13]. Also, nephrectomy-induced azotaemia and mild metabolic acidosis have been suggested as a possible biological explanation for survival benefit in these patients [14].

As evidence on the matter remains somewhat conflicting, there is a scarcity of tools for clinical decision-making considering CN and metastasectomies. We present a nationwide registry-based dataset of 732 consecutive patients diagnosed with primary mRCC from 2005 to 2010, a 6-year timeframe, which intersects the transition in medical treatment practices from cytokine-based to targeted therapies. Thus, we aimed to objectively evaluate the possible benefits gained by surgical treatment in a real-life, population-based setting.

## Materials and methods

All patients reported with synchronous mRCC or renal cell carcinoma (RCC) with unknown metastatic status diagnosed between 2005 and 2010 were identified from the Finnish Cancer Registry, which includes all new cancer cases in Finland. Based on these data, patient records of 2169 consecutively diagnosed patients were requested from the treating hospitals. Four hundred and ten cases were diagnosed outside the defined timeframe and thus excluded. Patients without evidence of mRCC at the time of diagnosis (n = 500) or missing treatment or follow-up details (n = 166) were ruled out, as well as patients under 18 years of age (n = 20) and with other cancer of advanced stage (n = 57). Thirty-one posthumously diagnosed cases were also excluded. Of the remaining 985 cases, considering the low number of ECOG 3–4 patients among the surgically treated population, and to reflect a more contemporary practice towards cytoreductive surgery for patients with low performance status, we limited the analysis for the ECOG 0–2 patient groups only. A total of 732 patients were included in the final analysis.

The following clinicopathologic variables were collected: sex, age at the time of diagnosis, primary cancer characteristics (T stage, Fuhrman grade, and histology), metastasis details (location of metastasis and number of metastatic sites), ECOG performance status, laboratory results (serum hemoglobin and C-reactive protein [CRP]) and cause of death. T stage was reassigned according to the 2017 TNM classification [15], and ECOG performance status at time of diagnosis was evaluated retrospectively by the author, if not clearly specified in the patient records.

Treatment protocols and follow-up were at the discretion of the treating physician. All patients received at least one dose of the medications that were considered in the analysis.

### Statistical analysis

The main outcome was OS. Survival time was calculated as the time from diagnosis to death or to the last follow**-**up contact. In this analysis**,** follow-up was limited to 10 years. The survival distribution and median survival were assessed with Kaplan-Meier estimates. Univariate associations between OS and baseline clinical and demographic factors were examined. Comparison of baseline characteristics and risk factors between the treatment subgroups was performed using the *χ*2 test. Log-rank tests were used to test the influence of baseline factors and treatments on OS. Significance was taken at p < 0.05. Hazard ratios (HRs) with 95% confidence intervals (95% CI) for possible significant prognostic factors and treatment options were calculated using univariable Cox regression. Time-dependent multivariable adjusted Cox regression model was used to preclude the immortal-time bias considering treatment options [16, 17]. Due to missing data on some of the risk factors, a multiple imputation technique was used to increase the precision and reduce bias in the analyses. Two different multivariable adjusted Cox models were created. In model 1, we imputed one-by-one all factors, that were found significant (p < 0.05) according to univariate analysis or, had a significant impact on Akaike’s Information Criterion, compared to the previous model. In model 2, we only used variables known before the decision of potential surgery. Furthermore, in model 2, a directed acyclic graph (DAG) was created to only include variables needed to get an unbiased adjusted estimate of treatment effect (HR). All analyses were performed using IBM SPSS version 27 (Chicago, IL, USA) or SAS version 9.4 (SAS Institute Inc., Cary, NC, USA).

## Results

### Patient characteristics

A total of 732 patients were included in the analysis, 424 (57.9%) were male and 308 (42.1%) were female. Median age was 67.0 years. Mean follow-up was 27.1 (range 0.3–120.0) months. Of the 548 (74.9%) patients with available histological diagnosis, 483 (88.1%) had clear cell histology and 80 (11.9%) had other histology. Sarcomatoid features were found in 47 (8.6%) of these patients. Histological diagnosis was unavailable for 184 (25.1%) patients. Patients identified with a local tumor stage included: T1, 131 (17.9%); T2, 109 (14.9%); T3, 328 (44.8%); and T4, 118 (16.1%). Additionally, T staging was not reliably defined for 46 (6.3%) patients. A more detailed distribution of baseline characteristics is shown in Table 1.

**Table 1:** Baseline patient characteristics in different nephrectomy status patient groups

	Number of patients (%)				
Baseline characteristics	Nephrectomy status				
	No nephrectomy	Cytoreductive nephrectomy	Surgery with curative intent	Total (n = 732)	^**1**^p value
**Total**	275 (37.6%)	389 (53.1%)	68 (9.3%)	732 (100.0%)	
**Gender**					0.28
Male	154 (56.0%)	235 (60.4%)	35 (51.5%)	424 (57.9%)	
Female	121 (44.0%)	154 (39.6%)	33 (48.5%)	308 (42.1%)	
**Age at diagnosis (years)**					
Median (25th–75th percentiles)	73.3 (61.9–80.0)	64.0 (57.7–72.6)	62.2 (57.3–72.1)	67.0 (59.6–77.6)	< 0.001
ECOG*					< 0.001
0	15 (5.5%)	41 (10.5%)	7 (10.3%)	63 (8.6%)	
1	116 (42.2%)	272 (69.9%)	50 (73.5%)	438 (59.8%)	
2	144 (52.4%)	76 (19.5%)	11 (16.2%)	231 (31.6%)	
T stage*					0.063
T1	64 (26.6%)	57 (15.1%)	10 (14.9%)	131 (19.1%)	
T2	48 (19.9%)	49 (13.0%)	12 (17.9%)	109 (15.9%)	
T3	66 (27.4%)	227 (60.1%)	35 (52.2%)	328 (47.8%)	
T4	63 (26.1%)	45 (11.9%)	10 (14.9%)	118 (17.2%)	
**N stage**					0.638
N0	161 (58.5%)	240 (61.7%)	39 (57.4%)	440 (60.1%)	
N1	114 (41.5%)	149 (38.3%)	29 (42.6%)	292 (39.9%)	
**Number of metastatic sites**					< 0.001
1	53 (19.3%)	114 (29.3%)	49 (72.1%)	216 (29.5%)	
2	75 (27.3%)	146 (37.5%)	16 (23.5%)	237 (32.4%)	
≥ 3	147 (53.5%)	129 (33.2%)	3 (4.4%)	279 (38.1%)	
**Metastatic sites**					
Distant lymph nodes	90 (32.7%)	99 (25.4%)	6 (8.8%)	195 (26.6%)	< 0.001
Lungs	192 (69.8%)	247 (63.5%)	17 (25.0%)	456 (62.3%)	< 0.001
Bone	96 (34.9%)	111 (28.5%)	6 (8.8%)	213 (29.1%)	0.001
Adrenal gland	53 (19.3%)	70 (18.0%)	17 (25.0%)	140 (19.1%)	0.398
Liver	75 (27.3%)	51 (13.1%)	5 (7.4%)	131 (17.9%)	< 0.001
Brain	24 (31.2%)	16 (18.6%)	2 (12.5%)	42 (23.5%)	0.093
Histology *					0.007
Clear cell carcinoma	90 (79.6%)	335 (90.5%)	58 (89.2%)	483 (88.1%)	
Other	23 (20.4%)	35 (9.5%)	7 (10.8%)	65 (11.9%)	
Hemoglobin < LLN*	132 (63.8%)	141 (54.0%)	28 (60.9%)	301 (58.6%)	0.099
**CRP > ULN**	132 (77.6%)	154 (72.3%)	26 (78.8%)	312 (75.0%)	0.424

^1^P value between nephrectomy status groups

*Histological diagnosis was missing for 184 patients, T stage for 46, hemoglobin for 218, and CRP for 316 patients. Percentages were only calculated for the group of patients for whom data on these variables were available

Note: *ECOG* = Eastern Cooperative Oncology Group, *LLN* = lower limit of normal, *CRP* = C-reactive protein, *ULN* = upper limit of normal

### Prognostic factors

In the univariate analysis, OS was significantly affected by the following prognostic factors: age, histology (clear cell vs. other), primary tumor T stage, ECOG performance status, number of metastatic sites, local N stage, distant lymph node metastases, bone metastases, liver, and brain metastases. For biochemical prognostic factors, increased serum CRP and hemoglobin less than the lower limit of normal (LLN) were also identified as statistically significant (Table 2).

**Table 2:** Prognostic factors for OS according to univariable Cox model

Prognostic factor	Hazard ratio (95% CI)	p value
**Age (years)**		
<60	1 (ref)	
60–68	1.01 (0.82–1.25)	0.89
69–77	1.30 (1.06–1.59)	**0.011**
> 78	2.09 (1.67–2.61)	**< 0.001**
**Local tumor stage**		
T1–3	1 (ref)	
T4	1.46 (1.19–1.79)	**< 0.001**
**ECOG**		
0	1 (ref)	
1	1.41 (1.06–1.88)	**0.018**
2	2.94 (2.18–3.98)	**< 0.001**
**Number of metastatic sites**		
1	1 (ref)	
2	1.37 (1.13–1.67)	**0.001**
≥ 3	1.79 (1.49–2.16)	**< 0.001**
**Local lymph node metastases**	1.29 (1.10–1.50)	**0.001**
**Distant lymph node metastases**	1.19 (1.01–1.41)	**0.041**
**Bone metastases**	1.21 (1.03–1.43)	**0.021**
**Liver metastases**	1.52 (1.25–1.84)	**< 0.001**
**Brain metastases**	1.84 (1.28–2.63)	**0.001**
Adrenal metastases	1.06 (0.88–1.29)	0.525
Lung metastases	1.10 (0.94–1.29)	0.225
**Histology**		
Clear cell carcinoma	1 (ref)	
Other	1.54 (1.18–2.00)	**0.001**
Not available	2.63 (2.20–3.14)	**< 0.001**
**CRP > ULN**	1.44 (1.14–1.82)	**0.002**
**Hemoglobin < LLN**	1.50 (1.25–1.80)	**< 0.001**

Note: *CI* = confidence interval, ref = reference group, *ECOG* = Eastern Cooperative Oncology Group, *CRP* = C-reactive protein, *ULN* = upper limit of normal, *Hb* = haemoglobin, *LLN* = lower limit of normal

There were significant differences in the accumulation of risk factors between the surgical treatment groups. The population in the non-nephrectomy group had a higher median age at the time of diagnosis, compared to the patients with primary tumors that were surgically removed. Also, in the non-surgically treated group, there was a remarkably greater proportion of patients with impaired (ECOG 2) performance status and multiple metastatic sites. However, no significant difference in serum hemoglobin or CRP levels was found between the groups. Nephrectomy was less often performed in patients with T4 tumors, compared to T1–3 tumors (46.6% vs. 68.7%, respectively; p < 0.001). Patients with T4 tumors were more likely to have anemia (73.9% vs. 54.8%; p = 0.001), compared to patients with T1–3 tumors. Detailed information on the distribution of patient characteristics in different nephrectomy status groups is shown in Table 1.

### Effect of nephrectomy and metastasectomies on survival

**Fig. 1:**
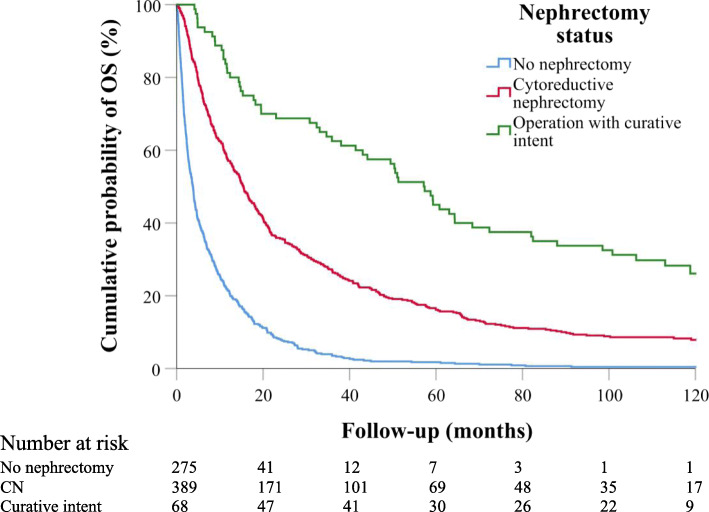
Kaplan-Meier curves of OS in patient groups with different nephrectomy status

Of all 732 analyzed cases, nephrectomy was performed in 457 (62.4%) patients. In 389 (53.1%) cases, the operation was cytoreductive, whereas nephrectomy and metastasectomies of all macroscopic metastatic lesions (surgery with curative intent) were performed on 68 (9.3%) patients. In the cytoreductive nephrectomy group, a concurrent adrenalectomy was performed for 26 of 70 patients with adrenal metastases. The median OS of all patients was 11.9 (10.4–13.3) months. The median OS for patients who underwent nephrectomy was 18.6 (95% CI = 15.9–21.2) months, which was higher compared to patients who did not undergo nephrectomy (5.9 [95% CI = 4.6–7.2] months, p < 0.001). Patients who had surgery with curative intent had a median OS of 51.3 (95% CI 36.0–66.6, P < 0.001) months, whereas patients who had a CN had a median OS of 16.6 (95% CI 14.2–19.1, P < 0.001) months. Kaplan-Meier estimates are shown in Fig. 1.

### Nephrectomy in patients receiving medical treatments

Medical treatments were mainly given after nephrectomy. Of the 349 patients who had both surgery and any medical treatments, 334 (95.7%) underwent nephrectomy before medication. In the CN group of 308 patients, surgery took place before medical treatments in 294 (95.5%) cases, and in 40 (97.6%) of 41 cases in the intended curative surgery group, respectively.

In the subgroup of 148 patients who had first-line interferon with or without concurrent chemotherapy as a first-line medical treatment, surgery with curative intent was performed in nine patients. CN was performed in 93 of these patients and 46 did not undergo nephrectomy. TT as a first-line medical treatment was administered for 314 patients, of whom 32 had surgery with curative intent, 210 underwent CN and 72 did not undergo nephrectomy. Of the 10 patients who only received cytotoxic chemotherapy as first-line medical treatment, no one was operated with curative intent. CN was performed on five patients and five had no surgical treatment. Kaplan-Meier overall survival estimates for the different combinations of surgical and first-line medical therapy are shown in Table 3.

**Table 3:** Cross tabulation of survival estimates in different surgical and first-line medical treatment groups

	First-line medical treatment				
Nephrectomy status		No surgical treatment	Cytoreductive nephrectomy	Surgery with curative intent	p value
		Median OS (95% CI), months			
	No medical treatment	3.5 (2.5–4.4)	4.1 (3.0–5.2)	50.6 (0.0–105.7)	< 0.001
	Chemotherapy	7.3 (5.4–9.2)	10.3 (3.4–17.3)	–	0.92
	Interferon ± chemotherapy	10.0 (7.3–12.7)	18.6 (14.7–22.4)	43.6 (0.0–116.8)	0.002
	Targeted therapy	10.6 (8.1–13.1)	21.9 (16.8–27.0)	57.2 (36.2–78.1)	< 0.001

Note: *OS* = overall survival, *CI* = confidence interval

As some of the patients received both interferon-based treatment with or without cytotoxic chemotherapy (mostly vinblastine) and also targeted therapy at some point of their treatment, the survival data between these groups were compared per nephrectomy status groups. Results are presented in Table 4.

**Table 4:** Survival estimates according to medical therapies and nephrectomy status

	Medical treatment					p value
Nephrectomy status		No nephrectomy (n)	Cytoreductive nephrectomy (n)	Curative intent (n)	Overall (n)	
		Median OS, months (95% CI) (n)				
	Interferon, no TT	9.5 (7.9–11.1) (41)	11.7 (5.1–18.3) (51)	17.8 (2.1–33.5) (7)	10.4 (8.1–12.7) (99)	0.043
	TT ± interferon	10.8 (8.4–13.3) (78)	25.1 (20.4–29.8) (253)	58.7 (47.3–70.2) (34)	21.3 (17.9–24.6) (373)	< 0.001
**p value**		0.15	0.004	0.296	< 0.001	

Note: *OS* = overall survival, *CI* = confidence intervals, *TT* = targeted therapy

As interferon-based therapy has been replaced by TT, and more recently also with immuno-oncologic therapies, we further analyzed the data excluding patients who received first-line interferon. For this subgroup, baseline patient characteristics and prognostic factors are presented in Supplemental Table 1 and Supplemental Table 2, respectively (see Additional file 1 and Additional file 2, respectively). In brief, the distribution of baseline characteristics between nephrectomy status groups were similar to Table 1, except for local T stage, where a statistically significant (p < 0.001) difference was found, unlike in the data with all patients (p = 0.063). According to univariate Cox regression analysis, the statistically significant risk factors for OS remained the same, except for distant lymph node metastases (p = 0.081 vs. p = 0.041 in the supplemental analysis vs. in the data with all patients, respectively). Survival analyses are presented in Additional file 3, and Kaplan-Meier estimates are shown in Supplemental Figure 1 (see Additional file 3). As for OS estimates, the observed differences between surgical therapy groups remained statistically significant in the concerned subgroup, as well as in the data with all patients.

### Multivariable-adjusted analyses

To evaluate the independent role of surgery to overall survival, and to eliminate immortality time bias from the estimates, a time-dependent Cox regression analysis was performed, adjusting for the following covariates (model 1): T4 tumor, ECOG, bone metastases, liver and brain metastases, and first-line medical therapy, as these variates were identified as significant prognostic factors in univariate Cox regression analysis (Table 2). To prevent bias due to the significant proportion of cases with no confirmed tumor histology in the non-surgically treated group, we ruled out histology as a covariate. Compared to patients who did not undergo nephrectomy, HR for death was 0.71 (95% CI = 0.59–0.86, p < 0.001) in the CN group, and 0.27 (95% CI = 0.19–0.37, p < 0.001) in the curatively operated group. Due to some missing values in the data concerning adjusting covariates, a multiple imputation (MI) model was applied to increase the accuracy of the model. In the MI model, HR for death was 0.73 (95% CI = 0.61–0.87, p < 0.001) in the CN group and 0.26 (95% CI = 0.19–0.36, p < 0.001) months in the curatively operated group.

To further evaluate the relevance of covariates for the multivariable analysis regarding the role of surgical treatment, apart from Cox regression univariate analysis for prognostic risk factors, a DAG was formulated. According to the DAG causal effect identification, age, ECOG, medical therapy, number of metastatic sites, and T4 tumor were identified as minimal sufficient adjustment sets for estimating the total effect of nephrectomy on OS. A time-dependent Cox regression analysis with the mentioned adjusting covariates was then performed. In this analysis, all relevant data was available for a total of 678 (92.6%) patients; HR for death was 0.72 (95% CI = 0.59–0.88, p = 0.001) in the CN group and 0.31 (95% CI = 0.22–0.44, p < 0.001) in the intended curative surgery group, compared to the patients who did not have a nephrectomy. In the MI model with the DAG-identified covariates, HR for death was 0.73 (95% CI = 0.61–0.88, p < 0.001) and 0.29 (95% CI = 0.21–0.40, p < 0.001) in the CN and curatively operated groups, respectively.

### Surgical complications/postoperative morbidity and mortality

Nephrectomy complications at 30-day surveillance according to the revised Clavien-Dindo classification [18, 19] are shown in Table 5. A total 30-day and 90-day complication rates were 18.4% and 20.4%, respectively. The grade 5 figures represent total postoperative mortality and include all deaths that occurred within the 30-day postoperative period, whether or not they seemed to be related to the operation. Data regarding 90-day follow-up complications are shown in Supplemental Table 3 (see Additional file 4).

**Table 5:** Overall rates of complications of 457 nephrectomies in 30-day follow-up

Clavien-Dindo grade						
Type of complication	Grade 1	Grade 2	Grade 3	Grade 4	Grade 5	Total
**Surgical site infection**	1 (0.2%)	1 (0.2%)	3 (0.7%)	–	–	5 (1.1%)
**Gastrointestinal**	3 (0.7%)	2 (0.4%)	9 (2.0%)	1 (0.2%)	2 (0.4%)	17 (3.7%)
**Vascular/hemorrhage**	–	5 (1.1%)	8 (1.8%)	2 (0.4%)	3 (0.7%)	18 (3.9%)
**Non-surgical infection**	1 (0.2%)	7 (1.5%)	–	–	4 (0.9%)	12 (2.6%)
**Comorbidity/disease progression**	1 (0.2%)	3 (0.7%)	3 (0.7%)	4 (0.9%)	4 (0.9%)	15 (3.3%)
**Thromboembolic**	3 (0.7%)	4 (0.9%)	2 (0.4%)	–	–	9 (2.0%)
**Other**	1 (0.2%)	1 (0.2%)	1 (0.2%)	–	–	3 (0.7%)
**Wound dehiscence/hernia**	2 (1.0%)	1 (0.2%)	2 (0.4%)	–	–	5 (1.8%)
**Total**	12 (2.6%)	24 (5.3%)	28 (6.1%)	7 (1.5%)	13 (2.8%)	84 (18.4%)

## Discussion

In this nationwide mRCC population, the median OS for patients who underwent nephrectomy was significantly higher compared to patients who did not undergo nephrectomy (18.6 vs. 5.9 months, respectively). Complete metastasectomy, in addition to radical nephrectomy, resulted in a significantly improved median OS of 51.3 months, compared to both non-surgically treated and CN groups. This adds to the current evidence concerning the advantages of metastasectomies in the treatment of mRCC, when all macroscopic tumors are extirpable [20–22]. In the multivariable adjusted analyses, we observed a statistically significant OS benefit gained by both CN and surgery with a curative purpose. This advantage was distinct in both statistical models with different sets of adjusting covariates. The OS was significantly higher in the CN group, compared to the non-surgically treated group, in both interferon and TT first-line medical therapy subgroups.

Survival rates in the study population and its subgroups were comparable to other population-based studies. In 2016, De Groot et al. reported a median OS of 17.9 months for patients with primary mRCC treated with CN and sunitinib, whereas in their study population, patients who received sunitinib but did not undergo CN, the median OS was significantly lower (8.8 months) [23]. Correspondingly, a Norwegian population-based study by Beisland et al. reported a median OS of 10.0 months and 8.0 months for primary mRCC patients diagnosed in 2009–2011 and 2006–2008, respectively [24]. In addition, similar results were shown in a Swedish population-based study, reflecting a median OS of mRCC patients diagnosed 2006–2008 as 12.4 months [25]. In the Danish nationwide DARENCA-2 study population consisting of biopsy-proven mRCC referred for medical oncologic treatment, the median OS for treated patients increased significantly from 11.5 months in 2006 to 17.2 months in 2010 [26]. These results correlated closely to those reported in our study. However, it must be noted that as our dataset consisted only of patients with primary (synchronous) mRCC, there may be a tendency towards more aggressive cancers, compared to datasets with both synchronous and metachronous mRCC [27, 28].

The superiority of TT over interferon-based therapy [3–5] was not approved in this cohort, when first-line medical therapies were compared. We detected no significant differences in median survival of patients treated with first-line TT compared to those treated with first-line interferon-based therapy, either in the no-nephrectomy population or after CN. However, the survival estimates for CN patients who received TT only, or in addition to interferon, were significantly higher than for CN patients who received interferon only. Such differences between OS in the aforementioned medical therapy regimes were not significant for patients who did not undergo nephrectomy or for those who underwent surgery with curative intent, although such trends can be observed, particularly in the latter group.

In our population-based study, more than 95% of the medically treated patients in the CN group underwent nephrectomy before medical treatment, and no analysis of the possible advantages or disadvantages of reciprocal sequencing could be made, owing to the limited number of sample cases. The sequencing of CN and the TKI sunitinib were investigated in a randomized trial by Bex et al. [29], and although no difference in the 28-week progression-free rate was demonstrated, a trend towards higher OS in the deferred CN arm was shown.

A considerable rate of severe complications occurred in the postoperative period. The complication rates are comparable to previous reports (i.e., Stang et al. reported an intrahospital mortality of 1.4% patients undergoing nephrectomy for the treatment of renal cancer in Germany in a 2-year period [30]). In a large contemporary cohort of 3644 CN-treated patients with mRCC from the USA, Palumbo et al. showed an overall complication risk of 39.7–55.3%, stratified according to age groups. Also, an increased risk of in-hospital mortality (3.6%) for older (≥ 71 years of age) patients was documented, compared to 1.7% and 1.0% in the age groups of 56–70 years and ≤ 55 years, respectively [31]. Our observed 30-day postoperative mortality was higher than in the recent REMARCC study, which reported 10 (1.3%) deaths and 45 (6.1%) high-grade complications from 736 CNs performed in 14 European institutes [32]. In our data, it is noteworthy that 6 of the 19 total grade 5 complications did not occur during the first 30-day postoperative period. Interestingly, one complication included the death of a patient during a prolonged observation period and the complications were not directly related to a certain perioperative surgical complication, but rather due to comorbidities or disease progression. In our study population, only a few operations were made with current mini-invasive approaches, as laparoscopic nephrectomy was not yet widely adopted in Finnish lower-volume hospitals during the studied period. This may contribute to the relatively high rate of postoperative morbidity and mortality, compared to more contemporary datasets [33]. Also, it must be noted that our results may underestimate the incidence of low-grade (Clavien-Dindo 1–2) complications, for the accuracy in reporting such episodes in patient reports may be incomplete in some institutions.

The strength of this study is that it is based on comprehensive national data from the Finnish Cancer Registry, which receives a notification from the treating hospitals of all suspected cancer cases [34]. To further complement this information, we examined the original patient records from the hospitals and combined these data to make our insight as detailed as possible. However, due to the retrospective and population-based nature of this investigation, all the baseline patient characteristics, such as performance status or laboratory values, could not be retrieved and were therefore not included in the analysis. As a result, patient distributions and analysis, according to the MSKCC or International Metastatic RCC Database Consortium (IMDC), risk groups could not be defined, which can be considered a limitation. Nevertheless, the risk factors for poor OS corresponded to those reported in earlier mRCC studies [35–40].

## Conclusion

Surgical treatment of mRCC is associated with a prominent OS benefit in patients with good and moderate performance status. Significant long-term responses can be achieved with total metastasectomy with curative intent, when feasible. CN was an independent predictor of improved OS in this nationwide database, including patients from both cytokine and TT eras. However, the importance of patient selection cannot be overemphasized, for cytoreductive nephrectomy is associated with relatively high complication rates and postoperative mortality.

## Supplementary Information

**Additional file 1: Supplemental Table 1.** Baseline patient characteristics in different nephrectomy status patient groups. 148 patients who received first-line interferon therapy with or without concurrent chemotherapy are not included.

**Additional file 2: Supplemental Table 2.** Prognostic factors for OS according to univariable Cox model. 148 patients who received first-line interferon therapy with or without concurrent chemotherapy are not included.

**Additional file 3: Supplemental Figure 1.** Survival analyses for different nephrectomy status groups excluding patients who received first-line interferon. Kaplan-meier curves are shown in Supplemental Figure 1.

**Additional file 4: Supplemental Table 3.** Overall rates of complications of 457 nephrectomies in 90-day follow-up.

## Data Availability

The patient data used in this study are not publicly available to preserve individuals’ privacy under the European General Data Protection Regulation.
