# Supplementary material for: Nephrectomy improves the survival of metastatic renal cell cancer patients with moderate to good performance status—results from a Finnish nation-wide population-based study from 2005 to 2010
Source: World J Surg Oncol. 2021 Jun 28;19:190. doi: 10.1186/s12957-021-02308-0 (PMC8240260; doi:10.1186/s12957-021-02308-0)
Supplement: Supplementary file 1 — Additional file 1: Supplemental Table 1. Baseline patient characteristics in different nephrectomy status patient groups. 148 patients who received first-line interferon therapy with or without concurrent chemotherapy are not included. [file 12957_2021_2308_MOESM1_ESM.docx]

Supplemental Table 1. Baseline patient characteristics in different nephrectomy status patient groups. 148 patients who received first-line interferon therapy with or without concurrent chemotherapy are not included.

|  | **Number of patients (%)** | | | |  |
| --- | --- | --- | --- | --- | --- |
| **Baseline Characteristics** | **Nephrectomy status** | | |  |  |
|  | **No nephrectomy** | **Cytoreductive nephrectomy** | **Surgery with curative intent** | **Total (n = 584)** | **^1^p-value** |
| **Total** | 229 (39.2 %) | 296 (50.7 %) | 59 (10.1 %) | 584 (100.0 %) |  |
| **Gender** |  |  |  |  | 0.196 |
| Male | 124 (54.1 %) | 181 (61.1 %) | 31 (52.5 %) | 336 (57.5 %) |  |
| Female | 105 (45.9 %) | 115 (38.9 %) | 28 (47.5 %) | 248 (42.5 %) |  |
| **Age at diagnosis (years)** |  |  |  |  |  |
| Median (25^th^–75^th^ percentiles) | 74.0 (62.2–81.2) | 64.9 (58.5–73.8) | 62.2 (57.5–71.9) | 68.0 (59.6–77.1) | < 0.001 |
| **ECOG*** |  |  |  |  | < 0.001 |
| 0 | 9 (3.9 %) | 34 (11.5 %) | 5 (8.5 %) | 48 (8.2 %) |  |
| 1 | 95 (41.5 %) | 199 (67.2 %) | 44 (74.6 %) | 338 (57.9 %) |  |
| 2 | 125 (54.6 %) | 63 (21.3 %) | 10 (16.9 %) | 198 (33.9 %) |  |
| **T stage*** |  |  |  |  | < 0.001 |
| T1 | 53 (26.5 %) | 46 (15.9 %) | 9 (15.5 %) | 108 (19.7 %) |  |
| T2 | 41 (20.5 %) | 40 (13.8 %) | 12 (20.7 %) | 93 (17.0 %) |  |
| T3 | 53 (26.5 %) | 167 (57.8 %) | 28 (48.3 %) | 248 (45.3 %) |  |
| T4 | 53 (26.5 %) | 36 (12.5 %) | 19 (15.5 %) | 98 (17.9 %) |  |
| **N stage** |  |  |  |  | 0.338 |
| N0 | 136 (59.4 %) | 188 (63.5 %) | 32 (54.2 %) | 356 (61.0 %) |  |
| N1 | 93 (40.6 %) | 108 (36.5 %) | 27 (45.8 %) | 228 (39.0 %) |  |
| **Number of metastatic sites** |  |  |  |  | < 0.001 |
| 1 | 45 (19.7 %) | 93 (31.4 %) | 44 (74.6 %) | 182 (31.2 %) |  |
| 2 | 64 (27.9 %) | 107 (36.1 %) | 13 (22.0 %) | 184 (31.5 %) |  |
| ≥3 | 120 (52.4 %) | 96 (32.4 %) | 2 (3.4 %) | 218 (37.3 %) |  |
| **Metastatic sites** |  |  |  |  |  |
| Distant lymph nodes | 78 (33.1 %) | 79 (26.7 %) | 4 (6.8 %) | 161 (27.6 %) | < 0.001 |
| Lungs | 155 (67.7 %) | 177 (59.8 %) | 11 (18.6 %) | 343 (58.7 %) | < 0.001 |
| Bone | 84 (36.7 %) | 88 (29.7 %) | 5 (8.5 %) | 177 (30.3 %) | < 0.001 |
| Adrenal gland | 42 (18.3 %) | 51 (17.2 %) | 15 (25.4 %) | 108 (18.5 %) | 0.333 |
| Liver | 66 (28.8 %) | 43 (14.5 %) | 5 (8.5 %) | 114 (19.5 %) | < 0.001 |
| Brain | 17 (26.6 %) | 11 (17.7 %) | 2 (13.3 %) | 30 (21.3 %) | 0.351 |
| **Histology *** |  |  |  |  | 0.004 |
| Clear cell carcinoma | 75 (77.3 %) | 253 (90.4 %) | 49 (87.5 %) | 377 (87.1 %) |  |
| Other | 22 (22.4 %) | 27 (9.6 %) | 7 (12.5 %) | 56 (12.9%) |  |
| **Hemoglobin < LLN*** | 112 (64.7 %) | 111 (54.7 %) | 24 (60.0 %) | 247 (59.4%) | 0.140 |
| **CRP > ULN** | 111 (79.3 %) | 112 (68.7 %) | 21 (77.8 %) | 244 (73.9 %) | 0.101 |

^1^P-value between nephrectomy status groups

*Histological diagnosis was missing for 151 patients, T stage for 37, hemoglobin for 168 and CRP for 254 patients. Percentages were only calculated for the group of patients for whom data on these variables were available.

Note: ECOG = Eastern Cooperative Oncology Group; LLN = lower limit of normal; CRP = C-reactive protein; ULN = upper limit of normal.
