# Supplementary material for: Nephrectomy improves the survival of metastatic renal cell cancer patients with moderate to good performance status—results from a Finnish nation-wide population-based study from 2005 to 2010
Source: World J Surg Oncol. 2021 Jun 28;19:190. doi: 10.1186/s12957-021-02308-0 (PMC8240260; doi:10.1186/s12957-021-02308-0)
Supplement: Supplementary file 2 — Additional file 2: Supplemental Table 2. Prognostic factors for OS according to univariable Cox model. 148 patients who received first-line interferon therapy with or without concurrent chemotherapy are not included. [file 12957_2021_2308_MOESM2_ESM.docx]

Supplemental Table 2. Prognostic factors for OS according to univariable Cox model. 148 patients who received first-line interferon therapy with or without concurrent chemotherapy are not included.

| **Prognostic factor** | **Hazard Ratio (95% CI)** | **p-value** |
| --- | --- | --- |
|  |  |  |
| **Age (years)** |  |  |
| < 60 | 1 (ref) |  |
| 60–68 | 0.95 (0.75–1.206) | 0.67 |
| 69–77 | 1.33 (1.06–1.68) | **0.014** |
| > 78 | 2.00 (1.57–2.56) | **< 0.001** |
| **Local tumour stage** |  |  |
| T1–3 | 1 (ref) |  |
| T4 | 1.61 (1.29–2.02) | **< 0.001** |
| **ECOG** |  |  |
| 0 | 1 (ref) |  |
| 1 | 1.46 (1.05–2.03) | **0.025** |
| 2 | 3.14 (2.22–4.43) | **< 0.001** |
| **Number of metastatic sites** |  |  |
| 1 | 1 (ref) |  |
| 2 | 1.45 (1.17–1.80) | **0.001** |
| ≥ 3 | 1.89 (1.53–2.32) | **< 0.001** |
| **Local lymph node metastases** | 1.33 (1.12–1.57) | **0.001** |
| **Distant lymph node metastases** | 1.18 (0.98–1.42) | 0.081 |
| **Bone metastases** | 1.26 (1.05–1.51) | **0.013** |
| **Liver metastases** | 1.54 (1.25–1.90) | **< 0.001** |
| **Brain metastases** | 1.95 (1.27–2.98) | **0.002** |
| Adrenal metastases | 1.02 (0.82–1.26) | 0.892 |
| Lung metastases | 1.18 (0.99–1.40) | 0.059 |
| **Histology** |  |  |
| Clear cell carcinoma | 1 (ref) |  |
| Other | 1.56 (1.17–2.09) | **0.002** |
| Not available | 2.81 (2.31–3.44) | **< 0.001** |
| **CRP > ULN** | 1.53 (1.19–1.98) | **0.001** |
| **Hemoglobin < LLN** | 1.51 (1.23–1.86) | **< 0.001** |

Note: CI = confidence interval; ref = reference group; ECOG = Eastern Cooperative Oncology Group; CRP = C-reactive protein; ULN = upper limit of normal; Hb = haemoglobin; LLN = lower limit of normal
