# Supplementary material for: Nephrectomy improves the survival of metastatic renal cell cancer patients with moderate to good performance status—results from a Finnish nation-wide population-based study from 2005 to 2010
Source: World J Surg Oncol. 2021 Jun 28;19:190. doi: 10.1186/s12957-021-02308-0 (PMC8240260; doi:10.1186/s12957-021-02308-0)

When patients who received first-line interferon therapy with or without concurrent chemotherapy were excluded, the median OS of all patients was 11.2 (9.6–12.8) months. The median OS for patients who underwent nephrectomy was 18.3 (95% CI = 15.3–21.5) months, which was higher compared to patients who did not undergo nephrectomy (4.7 [95% CI = 3.5–6.0] months, p < 0.001). Patients who had surgery with curative intent had a median OS of 57.2 (95% CI 46.6 – 67.7, P < 0.001) months, whereas patients who had a CN had a median OS of 15.6 (95% CI 12.4-18.8, P < 0.001) months. Kaplan-Meier estimates are shown in Supplemental Figure 1.

**Supplemental Fig. 1** Kaplan-Meier curves of OS in patient groups with different nephrectomy status. 148 patients who received first-line interferon therapy with or without concurrent chemotherapy are not included.


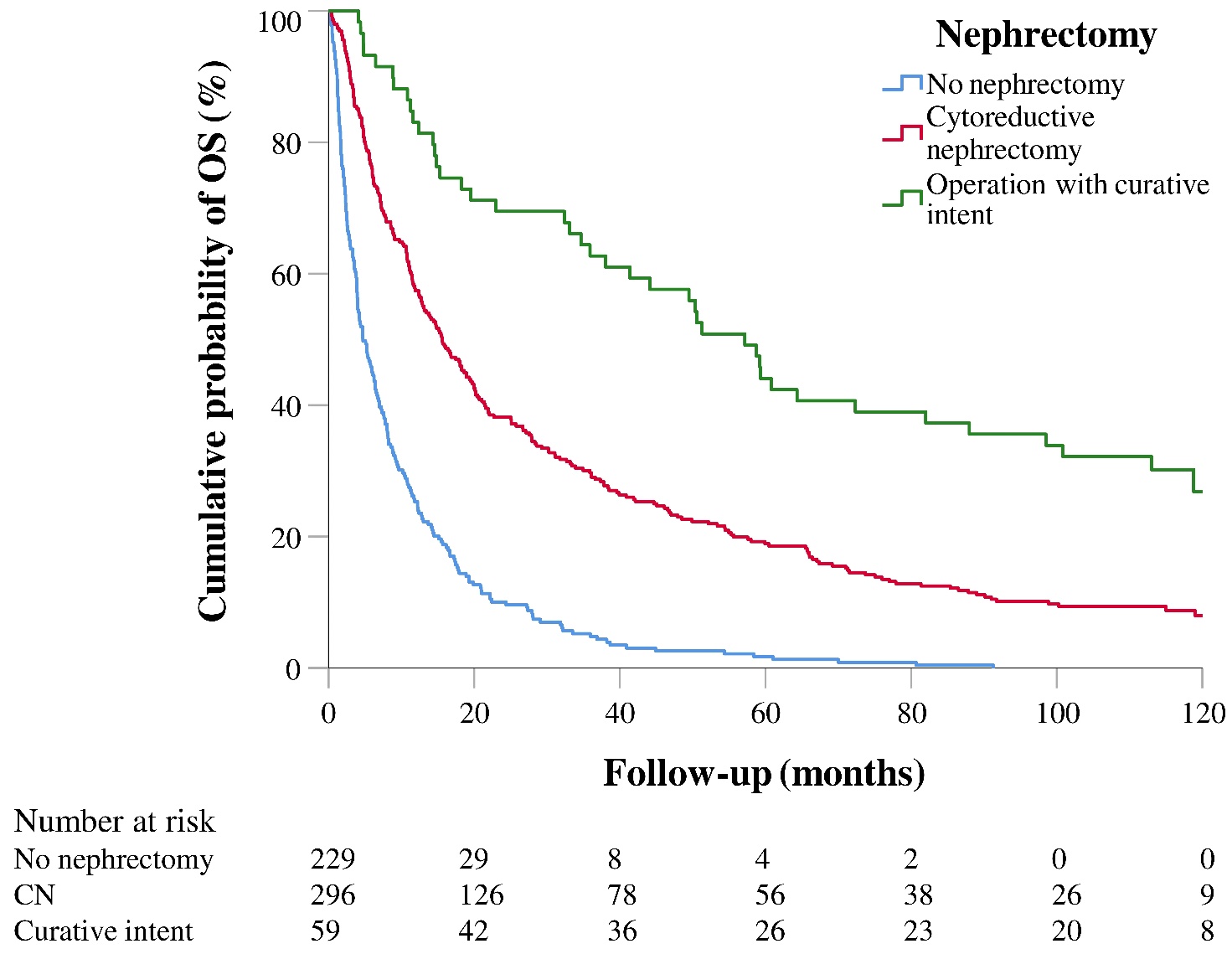

Supplement: Supplementary file 3 — Additional file 3: Supplemental Figure 1. Survival analyses for different nephrectomy status groups excluding patients who received first-line interferon. Kaplan-meier curves are shown in Supplemental Figure 1. [file 12957_2021_2308_MOESM3_ESM.docx]
