# Supplementary material for: Nephrectomy improves the survival of metastatic renal cell cancer patients with moderate to good performance status—results from a Finnish nation-wide population-based study from 2005 to 2010
Source: World J Surg Oncol. 2021 Jun 28;19:190. doi: 10.1186/s12957-021-02308-0 (PMC8240260; doi:10.1186/s12957-021-02308-0)
Supplement: Supplementary file 4 — Additional file 4: Supplemental Table 3. Overall rates of complications of 457 nephrectomies in 90-day follow-up. [file 12957_2021_2308_MOESM4_ESM.docx]

Supplemental Table 3. Overall rates of complications of 457 nephrectomies in 90-day follow-up.

| **Clavien-Dindo Grade** | | | | | | |
| --- | --- | --- | --- | --- | --- | --- |
| **Type of complication** | Grade 1 | Grade 2 | Grade 3 | Grade 4 | Grade 5 | Total |
| **Surgical site infection** | 1 (0.2%) | 1 (0.2%) | 3 (0.7%) | - | - | 5 (1.1%) |
| **Gastrointestinal** | 3 (0.7%) | 2 (0.4%) | 9 (2.0%) | 1 (0.2%) | 2 (0.4%) | 17 (3.7%) |
| **Vascular/hemorrhage** | - | 5 (1.1%) | 8 (1.8%) | 2 (0.4%) | 3 (0.7%) | 18 (3.9%) |
| **Non-surgical infection** | 1 (0.2%) | 7 (1.5%) | - | - | 4 (0.9%) | 12 (2.6%) |
| **Comorbidity/disease progression** | 1 (0.2%) | 3 (0.7%) | 3 (0.7%) | 4 (0.9%) | 10 (2.2%) | 21 (4.6%) |
| **Thromboembolic** | 3 (0.7%) | 4 (0.9%) | 2 (0.4%) | - | - | 9 (2.0%) |
| **Other** | 1 (0.2%) | 1 (0.2%) | 1 (0.2%) | - | - | 3 (0.7%) |
| **Wound dehiscence/hernia** | 5 (1.0%) | 1 (0.2%) | 2 (0.4%) | - | - | 8 (1.8%) |
| **Total** | 15 (3.3%) | 24 (5.3%) | 28 (6.1%) | 7 (1.5%) | 19 (4.2%) | 93 (20.4%) |
